# Cytokine profiling of exosomes derived from the plasma of HIV-infected alcohol drinkers and cigarette smokers

**DOI:** 10.1371/journal.pone.0201144

**Published:** 2018-07-27

**Authors:** Sunitha Kodidela, Sabina Ranjit, Namita Sinha, Carole McArthur, Anil Kumar, Santosh Kumar

**Affiliations:** 1 Department of Pharmaceutical Sciences, College of Pharmacy, University of Tennessee Health Science Center, Memphis, TN, United States of America; 2 Department of Oral and Craniofacial Science, School of Dentistry, University of Missouri-Kansas City, Kansas City, Missouri, United States of America; 3 Division of Pharmacology and Toxicology, School of Pharmacy, University of Missouri-Kansas City, Kansas City, Missouri, United States of America; University of Texas Medical Branch at Galveston, UNITED STATES

## Abstract

Cytokines and chemokines circulate in plasma and may be transferred to distant sites, via exosomes. HIV infection is associated with dysregulation of cytokines and chemokines, which subsequently contribute to the pathogenesis of HIV. Alcohol and tobacco exposure, which are prevalent in HIV-infected individuals, may induce changes in the expression of cytokines and chemokines. Therefore, our aim in this study was to quantify plasma exosomal cytokines and chemokines that we expect to exacerbate toxicity or disease progression in HIV-positive drug abusers. We measured the levels of cytokines and chemokines in the plasma and plasma exosomes of 39 patients comprising six groups: HIV-negative and HIV-positive non drug abusers, HIV-negative and HIV-positive alcohol users, and HIV-negative and HIV positive tobacco smokers. We measured six cytokines (TNF-α, IL-1β, IL-8, IL-6, IL-1ra, IL-10) and two chemokines (MCP-1 and RANTES). All were present in exosomes of healthy subjects, but their levels varied between different study groups. HIV-positive alcohol drinkers had higher levels of plasma IL-8 compared to those of HIV-positive non-drinkers. The IL-1ra level was significantly higher in exosomes of non-HIV-infected alcohol drinkers compared to those of HIV-positive alcohol drinkers. Interestingly, the IL-10 level was higher in exosomes compared with their respective plasma levels in all study groups except HIV-positive non-alcohol drinkers. IL-10 was completely packaged in exosomes of HIV-positive smokers. HIV-positive smokers had significantly higher levels of plasma IL-8 compared with HIV-positive non-smokers and significantly higher exosomal IL-6 levels compared with HIV-negative subjects. HIV-positive smokers had significantly increased plasma levels of IL-1ra compared to HIV-positive non-smokers. The MCP-1 levels in the plasma of HIV-positive smokers was significantly higher than in either HIV-positive non-drug abusers or HIV-negative smokers. Overall, the findings suggest that plasma cytokines and chemokines are packaged in exosomes at varying degrees in different study groups. Exosomal cytokines and chemokines are likely to have a significant biological role at distant sites including cells in the brain.

## Introduction

Abuse of alcohol and tobacco is prevalent among HIV-infected individuals. In the USA, the percentage of alcohol and tobacco use among HIVinfected individuals are 40% [1] and 42% [2], respectively. Systematic reviews on studies conducted among Africans strongly suggest an association between alcohol use and HIV infection in those populations [3–5]. Alcohol increases the risk of infection and also exacerbates HIV replication [6–9]. Moreover, alcohol reduces adherence to antiretroviral therapy (ART) and decreases ART efficacy, which could further increase HIV replication [10–12]. Similarly, smoking increases HIV replication by multiple mechanisms[13,14]. *In vivo* and *in vitro* studies demonstrate that tobacco smoking is associated with decreased immune response [15,16], increased inflammation [17] and oxidative stress [18–21], and increased occurrence of opportunistic infections [22,23]. In addition, pharmacokinetic interactions between smoking and ART drugs decrease the efficacy of ART, which could lead to progression of AIDS [24].

HIV infection is associated with chronic immune activation [25,26] and dysregulation of cytokines and chemokines, which subsequently contribute to the pathogenesis of HIV. Interactions between immune cells occur by direct cell-cell contact or through cytokine secretion. These cytokines are also likely to be circulated in plasma and transferred to other distant cells via exosomes. Exosomes, small extracellular vesicles (<200 nm), have recently been recognized as extremely valuable targets for biological research [27,28]. Their semi-selective ability to package and transport diverse biological cargos such as proteins, mRNAs, micro RNAs, and small molecules allow them to serve both as diagnostic biomarkers of disease states [29–32], and as potential therapeutic targets [29,33]. Konadu et al. have shown that the exosomes of HIV-seropositive patients have higher cytokine levels than those of HIV-seronegative individuals [30]. The expression of these cytokines may vary in the case of alcohol or tobacco exposure since the plasma cytokine levels of HIV-positive drug abusers are significantly different from HIV-positive non-abusers. However, cytokine levels in the plasma-derived exosomes of HIV-positive alcohol and tobacco abusers have not been studied. Moreover, studying the cytokine levels in exosomes is important, because the exosomal contents are more stable than freely circulating molecules and can have long-lasting effects [34]. A recent study from our lab has shown that exosomes derived from macrophages alter cytotoxicity and HIV replication when exposed to naïve macrophages [35]. As the HIV-induced immunomodulation might be altered by substance abuse (e.g. alcohol and tobacco), there is a need to identify a physiological marker to indicate the immune status of HIV patients with drug abuse disorders. We hypothesize that alcohol and tobacco use alter the expression of specific cytokines in exosomes in the plasma of HIV patients, that may be associated with enhanced toxicity and disease progression in HIV-positive drug abusers.

## Materials and methods

### Study design and subjects

We conducted a cross-sectional study consisting of 39 subjects after approval of the Institutional Review Board from the University of Missouri-Kansas City, in Kansas City, MO and the Institutional Ethics Committee from the Provincial Regional Hospital of the Ministry of Public Health in Bamenda, Cameroon. Written informed consent was obtained from all study participants and their medical history was collected during the recruitment. The study subjects belong to six different groups: HIVnegative non-drug abusers, (n = 10), HIV-positive non-drug abusers (n = 5), HIV-negative alcohol drinkers (n = 6), HIV-positive alcohol drinkers (n = 3), HIV-negative tobacco smokers (n = 11), and HIV-positive tobacco users (n = 4). Subjects were recruited as described in our earlier studies [9,14]. Briefly, patients between the ages of 20–65 years were recruited. The inclusion criteria for tobacco smokers were for those who smoke at least one pack of cigarettes per day for ≤ 20 years (mild-to-moderate tobacco users), and, for alcohol drinkers, those who drink 7–14 drinks/week for men and 4–7 drinks/week for women for the previous 6 months (mild-to-moderate alcohol users). The CD4 count (cells/μl) of HIV-positive non-drug abusers, HIV-positive tobacco users, and HIV-positive alcohol users ranged between 256–551, 412–584, and 321–480, respectively. The exclusion criteria of our study population were as described previously [9,14].

### Exosome isolation and characterization

We isolated exosomes from plasma using an exosome precipitation kit (Invitrogen). Briefly, plasma exosomes from study subjects were isolated by filtering plasma through a 0.22 μm filter to remove large vesicles (>200 nm) followed by precipitation using the exosome isolation kit (Invitrogen). Fifty μl of plasma from each subject was centrifuged at 2,000 g for 20 min followed by at 10,000 g for 20 min to remove any cell debris. The supernatant was transferred to a new tube and 0.5 volumes of PBS was added to it, followed by addition of 0.2 volumes of exosome precipitation reagent. The reaction mixture was incubated at room temperature for 10 min. After incubation, the sample was centrifuged at 10,000 g for 5 min, which resulted in the pure exosome pellet. We then characterized the size and zeta potential of exosomes by dynamic light scattering using a Zetasizer Nano-ZS (Malvern Instruments Inc, Malvern, UK) as described (31,35). We also characterized the exosomes for expression of the exosomal marker proteins CD63, CD81, and/or Alix by Western blotting. The size, shape, and quality of exosomes was characterized by by transmission electron microscopy with a JEOL 2000EXII (The Neuroscience Institute, University of Tennessee Health Science Center) as described previously [31,35]. We also quantified exosomes by measuring acetylcholinesterase (AchE) activity using a validated Amplex® Red Acetylcholine/Acetylcholinesterase Assay Kit (Molecular Probes, Invitrogen). Briefly, the exosome pellet was suspended in 100 μl of 50 mM Tris-HCl (pH 8.0) and incubated with 100 μL of the Amplex Red reagent containing 2 U/mL HRP, 0.2 U/mL choline oxidase, and 100 μM acetylcholine in a final volume of 200 μl. An aliquot of 100 μl (0.2 U/mL) acetylcholinesterase solution was used as positive control, and 100 μl 10 μM H_2_O_2_ working solution was used as second positive control. The reaction plate was incubated for more than 1 hour at room temperature in the dark. Fluorescence was measured every 15 min at λ_ex_ and λ_em_ of 530 nm and 590 nm, respectively. The optimal activity of AchE was observed at 45 min. The concentration of AchE in exosomes was between the ranges of 0.2–0.4 U/L/μg.

### Cytokine measurement

The protein levels of various cytokines and chemokines such as pro-inflammatory: TNF-α, IL-1β, IL-8, IL-6; anti-inflammatory: IL-1ra, IL-10; and chemokines: MCP-1, and RANTES in plasma and exosomes were measured using validated human Bio-Plex Pro Assays (Bio-RAD, CA, USA). Exosomes and plasma samples were diluted with Bio-Plex sample diluent and samples were prepared for the cytokine analysis according to the manufacturer’s instructions. The concentration (pg/ml) of eight different cytokines and chemokines were measured using a Luminex 200 ^TM^ system, and the data were analyzed using xPONENT^®^ software (S1 Dataset).

### Statistical analysis

The concentrations of cytokines and chemokines were presented as median and inter-quartile range. The Mann–Whitney U test was used to compare between plasma samples and their respective exosomes, as well as, among plasma and exosomes of different groups. The data were analyzed using Graph-Pad Prism version 5.00 (GraphPad Software, La Jolla, CA, USA). A P-value of ≤ 0.05 was considered statistically significant.

## Results

### Comparison of plasma and exosomal cytokines and chemokines between HIV-negative non-drug abusers, HIV-positive, HIV-negative drinkers, and HIV-positive drinker subjects

#### Pro-inflammatory cytokines

**Fig 1:**
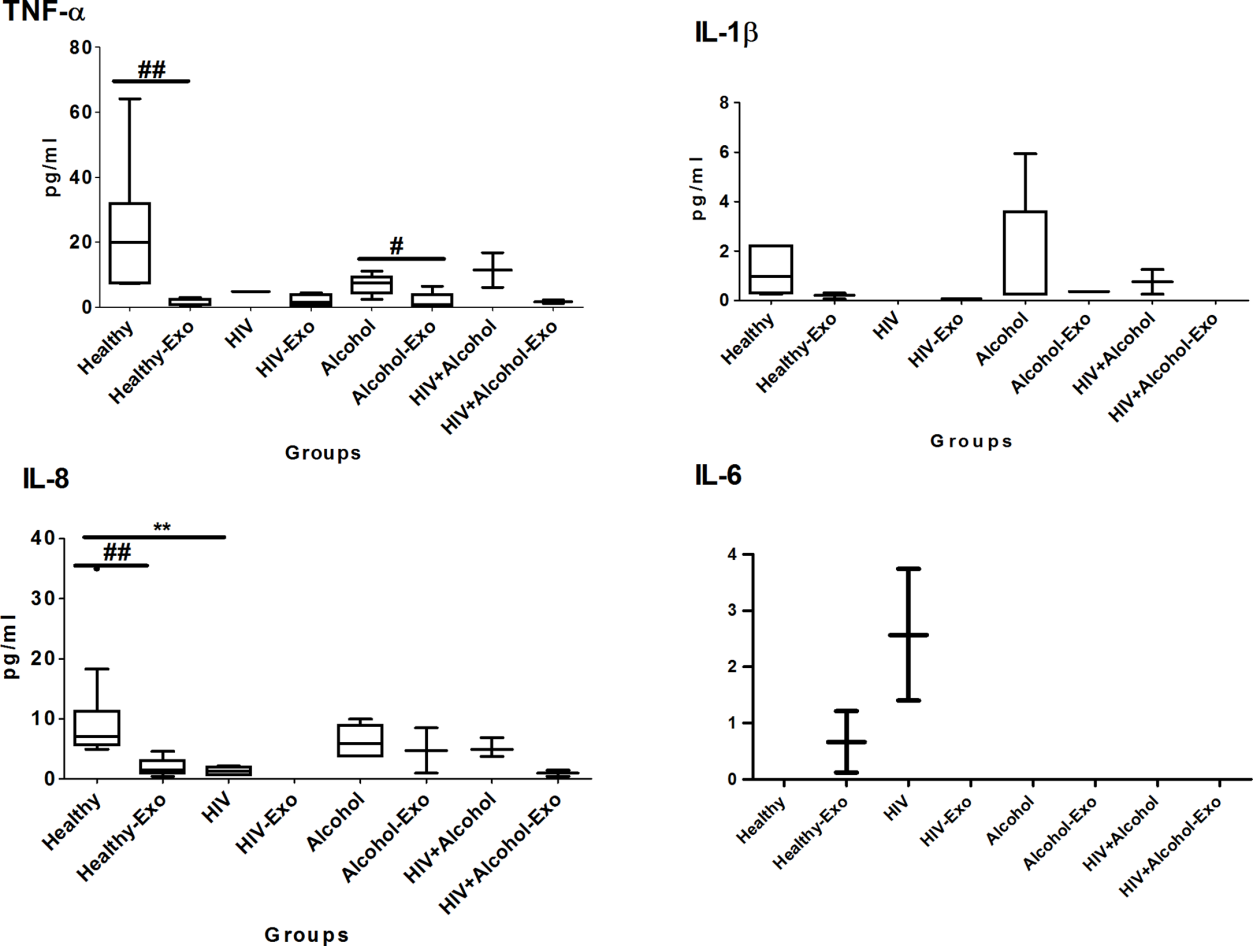
Box and whisker plots of pro-inflammatory cytokines between HIV-negative non-drug abusers (healthy), HIV-positive non-drug abusers (HIV), HIV-negative alcohol drinkers (alcohol), and HIV-positive alcohol drinker (HIV+alcohol) subjects. The box represents the 25^th^-75^th^ quartile, the whiskers represent the range of values, the median is presented as a line inside the box, and the out–of-range values are presented as circles. Mann–Whitney U test was used to compare between the plasma and their respective exosomes of different study groups, and significant p-values are represented as #(p≤0.05) and ##(p≤0.01). Comparison of cytokine levels among plasma as well as exosomes of different groups were done using Mann–Whitney U test, and significant p-values are given as * (p≤0.05), ** (p≤0.01) and $(p≤0.05), $ $((p≤0.01), respectively.

In general, the plasma pro-inflammatory cytokine levels in all the groups were higher than their respective exosomal cytokine levels (Fig 1 and Table 1). The relative percentages of plasma TNF-α, IL-1β, IL-8, and IL-6 packaged in exosomes of healthy subjects were 6%, 15%, 18%, and 0.66% respectively. TNF-α, IL-1β, and IL-8 were lower in HIV-positive subjects than HIV-negative subjects and similar to those obtained in alcohol drinkers. Although the level of IL-1β was higher in plasma samples of most groups than their respective exosomes, it was absent in plasma, but detectable in exosomes, of HIV subjects. IL-8 was not detectable in the exosomes of HIV patients. There was a significant difference in IL-8 levels between the plasma samples of HIV-negative and HIV-infected subjects. Further, IL-8 level were higher in plasma samples of HIV-positive drinkers than HIV-positive non-alcohol drinkers. The IL-6 was non-detectable in plasma samples of all the study groups except the HIV- positive group (Fig 1 and Table 1). Moreover, IL-6 was packaged in exosomes of HIV-negative subjects only, and was absent in other studied groups.

**Table 1:** Comparison of cytokine and chemokine levels between HIV-negative and-positive alcohol users.

Cytokine		HIV-negative non-drug abusers (N = 10)	HIV-positive (N = 5)	p-value	HIV-negative alcohol users(N = 6)	p-value	HIV-positive drinkers (N = 3)	p-value	p-value^b^	p-value^c^
**Pro-inflammatory**										
TNF-α	Plasma	20 (7.4–32)	4.8^a^	NA	7.4 (4.3–9.3)	0.08	11 (6.1–16)	0.35	NA	0.29
	Exosome	0.8 (0.7–2)	1.5(0.5–4)	0.77	0.79 (0.51–4.0)	0.80	1.7 (1.2–2.2)	0.66	0.85	0.39
	**p-value**	0.001	NA		0.02		0.10			
IL-1β	Plasma	0.97 (0.31–2.2)	ND	NA	0.26 (0.19–2.4)	0.58	0.75 (0.26–1.3)	0.65	NA	0.99
	Exosome	0.20 (0.06–0.31)	0.06^a^	NA	0.36 (0.36–0.36)	NA	ND	NA	NA	NA
	**p-value**	0.07	NA		NA		NA			
IL-8	Plasma	7.0 (5.6–11)	1.3 (0.64–2.0)	0.002	5.8 (3. 8–8.9)	0.35	4.9 (3. 80–6.90)	0.14	0.035	0.76
	Exosome	1.5 (0.94–3.0)	ND	NA	4.7 (0.94–8.5)	0.36	0.95 (0.40–1.46)	0.35	NA	0.40
	**p-value**	0.0007	NA		0.76		0.10			
IL-6	Plasma	ND	2.6 (1.4–3.7)	NA	ND	NA	ND	NA	NA	NA
	Exosome	0.67 (0.12–1.2)	ND	NA	ND	NA	ND	NA	NA	NA
	**p-value**	NA	NA		NA		NA			
**Anti-inflammatory**										
IL-1ra	Plasma	93.0 (36.2–128)	41 (35–73)	0.42	139 (105–193)	0.08	90 (65–98)	0.93	0.23	0.15
	Exosome	42 (9.4–49)	9.4 (3.0–9.4)	0.06	23 (15–53)	0.88	9.4 (3.4–12)	0.10	0.64	0.02
	**p-value**	0.10	0.03		0.008		0.10			
IL-10	Plasma	1.1 (0.61–1.7)	0.61 (0.11–3.3)	0.82	ND	NA	ND	NA	NA	NA
	Exosome	1.4 (0.75–1.9)	ND	NA	0.59 (0.06–3.3)	0.60	1.1 (0.83–1.4)	NA	NA	0.60
	**p-value**	0.53	NA		NA		NA			
**Chemokines**										
RANTES	Plasma	7052 (2852–15233)	13365 (8947–15416)	0.37	6973 (821.4–10500)	0.56	7262 (4843–25638)	0.69	0.57	0.71
	Exosome	530.8 (320.2–1744)	232 (149–871)	0.37	698.6 (327.7–2509)	0.71	706.8 (593.5–4118)	0.28	0.39	0.71
	**p-value**	0.001	0.007		0.09		0.10			
MCP-1	Plasma	31.4 (19.3–87.2)	139 (5.0–23)	0.02	36 (25–69)	0.58	35 (24–99)	0.57	0.07	0.99
	Exosome	3. 8 (1.5–4.7)	0.74^a^	NA	3.2 (2.4–13)	0.56	5.8 (3.4–6.9)	0.21	NA	0.51
	**p-value**	0.0001	NA		0.02		0.10			

Values are expressed as median (25^th^-75^th^ inter-quartile range); a-detectable only in one sample, b-Comparison between HIV- positive non-alcohol drinkers and HIV-positive alcohol groups, c-Comparison between HIV-negative alcohol drinkers and HIV-positive alcohol drinkers, ND-not detectable; NA-not applicable

#### Anti-inflammatory cytokines

**Fig 2:**
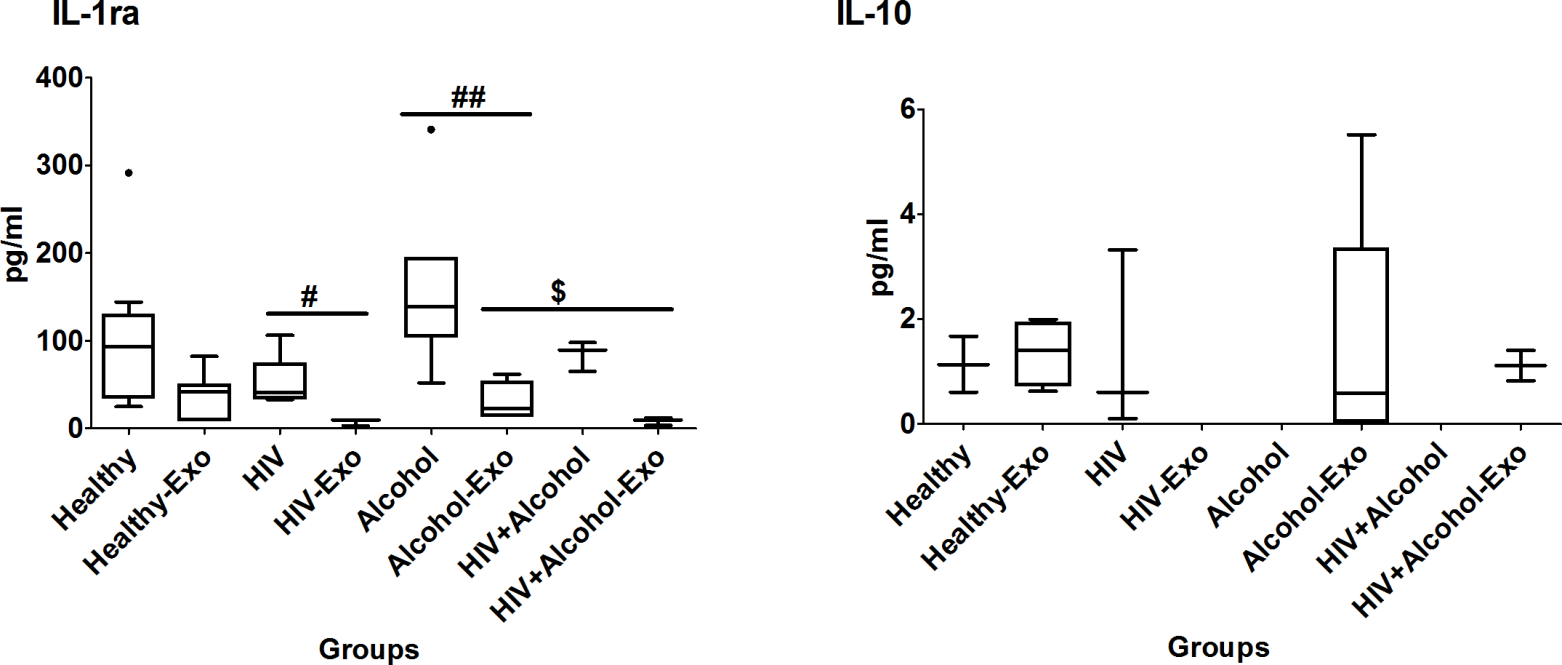
Box and whisker plots of anti-inflammatory cytokines between HIV-negative (healthy) non-drug abusers, HIV-positive non-drug abusers (HIV), HIV-negative alcohol drinkers (alcohol) and HIV-positive alcohol drinkers (HIV+alcohol). The box represents the 25^th^-75^th^ quartile, the whiskers represent the range of values, the median is presented as a line inside the box, and the out-of-range values are presented as circles. The Mann–Whitney U test was used to compare between plasma and exosomes of different study groups, and significant p-values are represented as # (p≤0.05), and ## (p≤0.01). Comparison of cytokine levels among plasma as well as exosomes of different groups were done using Mann–Whitney U test and significant p-values are given as * (p≤0.05), ** (p≤0.01) and $ (p≤0.05), $ $(p≤0.01), respectively.

The percentage of plasma IL-ra packaged in exosomes of HIV-negative subjects was relatively high at 39%. The IL-1ra level was detectable in both the plasma and exosomes of all of the studied groups, and, as expected, the level was relatively lower in exosomes than the respective plasma samples. The IL-1ra level was significantly higher in exosomes of HIV-negative alcohol users compared to those of HIV-positive drinkers, and to some extent compared to HIV-positive non-drinkers. Interestingly, the IL-10 level was high in exosomes compared to their respective plasma samples of all study groups except HIV-positive non-drinkers. Further, IL-10 was completely packaged in exosomes of alcohol drinkers and HIV-positive drinkers, and was not detected in their plasma samples. (Fig 2 and Table 1).

#### Chemokines

**Fig 3:**
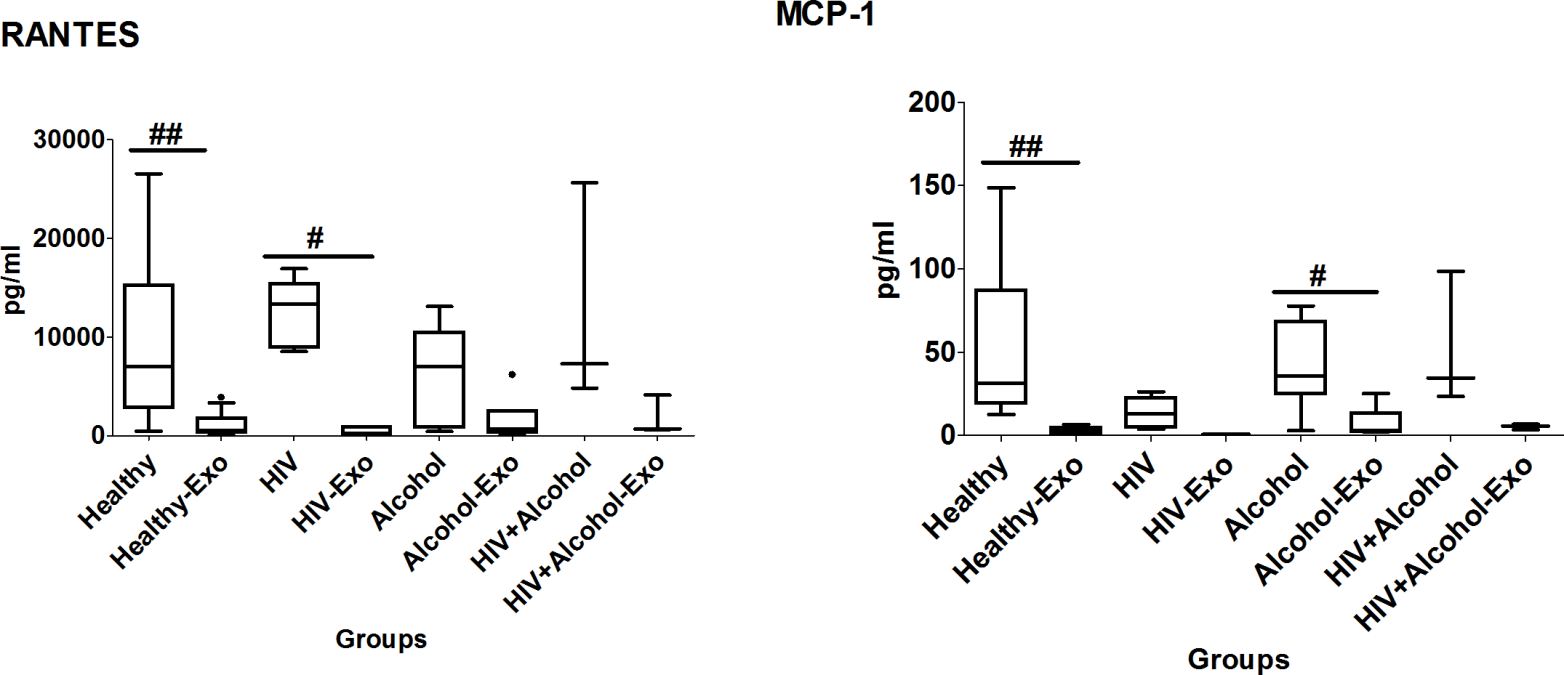
Box and whisker plots of anti-inflammatory cytokines between HIV-negative non-drug abusers (healthy), HIV-positive non-drug abusers (HIV), HIV-negative alcohol users (alcohol) and HIV-positive alcohol users (HIV+alcohol). The box represents the 25^th^-75^th^ quartile, the whiskers represent the range of values, the median is presented as a line inside the box, and the out-of-range values are presented as circles. The Mann–Whitney U test was used to compare between plasma and exosomes of different study groups and significant p-values are represented as #-(p≤0.05) and ##-(p≤0.01). Comparison of cytokine levels among plasma as well as exosomes of different groups were done using Mann–Whitney U test and significant p-values are given as * (p≤0.05), ** (p≤0.01) and $ (p≤0.05), $ $ (p≤0.01), respectively.

Relative to plasma, 12% of RANTES and 7% of MCP-1 were packaged in exosomes in uninfected non-drug abusers. Similar to cytokines (Figs 1 and 2), in general, the chemokine levels in exosomes were lower than those of respective plasma samples (Fig 3). More specifically, RANTES level was significantly higher in plasma than exosomes of healthy and HIV-positive subjects, while MCP-1 level was higher in plasma than exosomes of HIV-negative non-drug abusers and HIV-negative alcohol drinkers. Though not statistically significant, there is a pattern of relatively increased median levels of RANTES, but decreased median levels of MCP-1 in HIV subjects, compared with other groups.

### Comparison of plasma and exosomal cytokine andchemokine levels between HIV-negative non-drug abusers, HIV-positive, HIV-negative smokers, and HIV-positive smoker subjects

#### Pro-inflammatory cytokines

**Fig 4:**
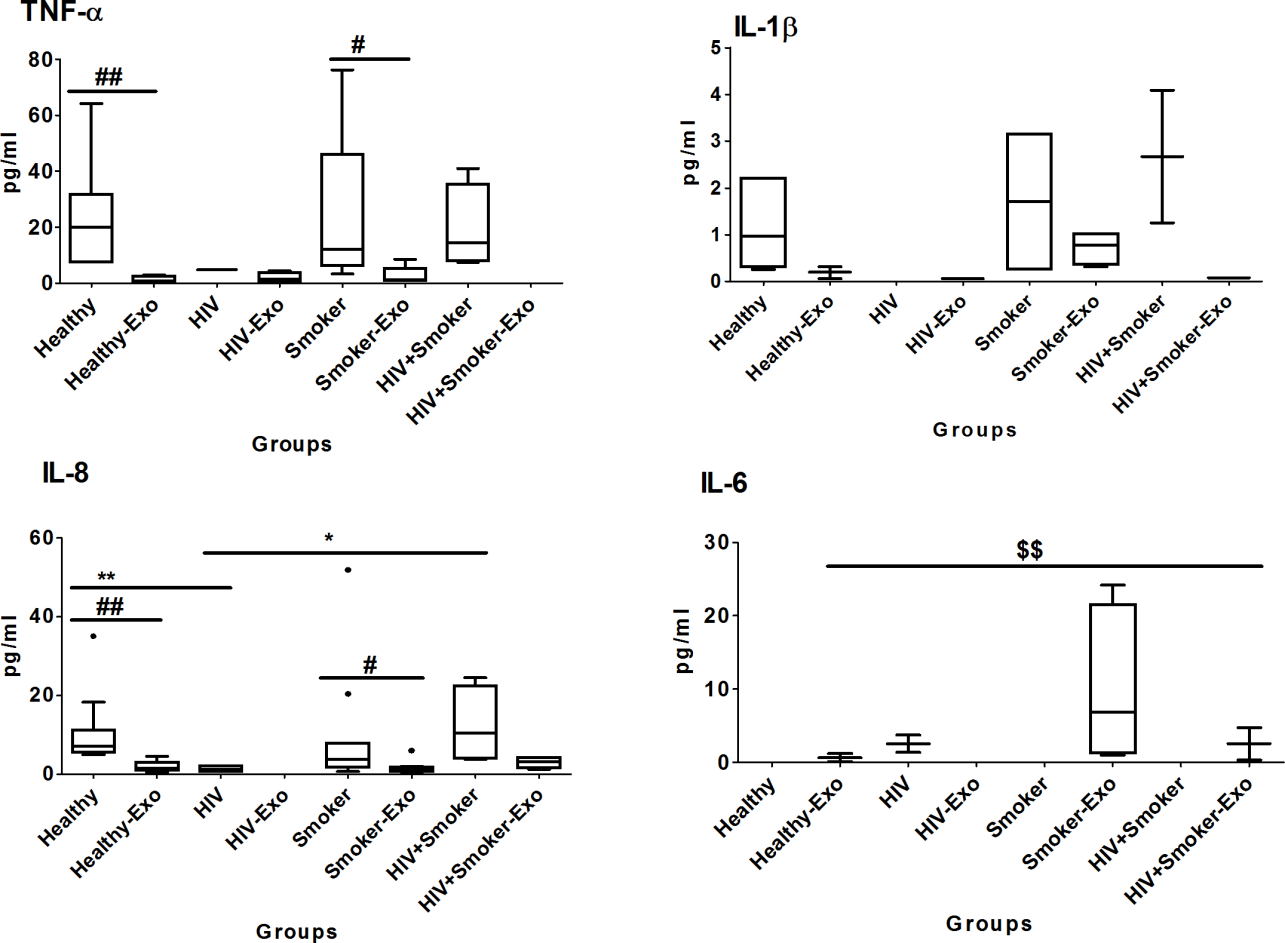
Box and whisker plots of pro-inflammatory cytokines between HIV-negative non-drug abusers (healthy), HIV-positive non-drug abusers (HIV), HIV-negative smokers (smokers) and HIV-positive smoker (HIV+smoker). For comparison with other study groups, the bar graphs for healthy and HIV subjects were replicated from Fig 1. The box represents the 25^th^-75^th^ quartile, the whiskers represent the range of values, the median is presented as a line inside the box, and the out-of-range values are presented as circles. The Mann–Whitney U test was used to compare between plasma and exosomes of different study groups and significant p-values are represented as # (p≤0.05) and ## (p≤0.01). Comparison of cytokine levels among plasma as well as exosomes of different groups were done using the Mann–Whitney U test and significant p-values are given as * (p≤0.05), ** (p≤0.01) and $ (p≤0.05), $ $ (p≤0.01), respectively.

Similar to the HIV-negative and HIV-positive non-drug abusers, the levels of all the pro-inflammatory cytokines were relatively lower in exosomes than their respective plasma samples of HIV-positive and -negative smokers, especially TNF-α in HIV+positive smokers, which was undetectable in exosomes. HIV-positive smokers also had significantly higher levels of plasma IL-8 than HIV-positive non-smokers (Fig 4, Table 2). Interestingly, IL-6 was packaged in exosomes of all the study groups except the HIV-positive non-drug abusers, even though IL-6 was not detectable in the plasma of the healthy, smoker, and HIV-positive smoker groups. The HIV-positive smokers had significantly higher exosomal IL-6 levels than those of HIV-negative subjects (Fig 4, Table 2).

**Table 2:** Comparison of cytokine and /chemokine levels between HIV-negative subjects, HIV-positive non-drug abusers, HIV-negative smokers, and HIV-positive non-smokers.

Cytokine		HIV-negative non-drug abusers (N = 10)	HIV-positive non-drug abusers (N = 5)	p-value	HIV-negative Smoker (N = 11)	p-value	HIV-positive smoker (N = 4)	p-value	p-value ^b^	p-value ^c^
**Pro-inflammatory**										
TNF-α	Plasma	20 (7.4–32)	4.8^a^	NA	12 (6.3–46)	0.99	14 (8.0–35)	0.99	NA	0.91
	Exosome	0.81 (0.69–2.5)	1.5 (0.49–3. 9)	0.77	1.1 (0.81–5.3)	0.41	ND	NA	NA	NA
	**p-value**	0.001	NA		0.01		NA			
IL-1β	Plasma	0.97 (0.31–2.2)	ND	NA	1.7 (0.25–3.2)	0.90	2. 72 (1.2–4.1)	0.13	ND	0.58
	Exosome	0.20 (0.06–0.31)	0.064^a^	NA	0.78 (0.37–1.0)	0.07	0.080^a^	NA	NA	NA
	**p-value**									
IL-8	Plasma	7.1(5.6–11)	1.3(0.64–2.0)	0.002	3.7 (1.7–7. 9)	0.06	10 (4.0–22)	0.72	0.01	0.21
	Exosome	1.5 (0.95–3.0)	ND	NA	1. 5 (0.69–1.8)	0.74	3.3 (1.5–4.2)	0.29	NA	0.16
	**p-value**	0.0007	NA		0.02		0.11			
IL-6	Plasma	ND	2. 6 (1.4–3.7)	NA	ND	NA	ND	NA		
	Exosome	0.67(0.12–1.2)	ND	NA	6.8 (1.3–21)	0.19	2.5 (0.35–4.7)	0.0001	NA	0.38
	**p-value**	NA	NA		NA		NA			
**Anti-inflammatory**										
IL-1ra	Plasma	93.0 (36.1–128)	41 (35–73)	0.42	38.6 (23.7–193)	0.45	156 (82.3–231)	0.18	0.03	0.17
	Exosome	42.2 (9.4–50)	9.4 (3.0–9.4)	0.05	16 (6.3–31)	0.09	31 (12–42)	0.64	0.07	0.35
	**p-value**	0.10	0.03		0.03		0.05			
IL-10	Plasma	1.1 (0.61–1. 7)	0.61 (0.11–3.3)	0.82	1. 7 (0.11–2.1)	0.82	ND	NA	NA	NA
	Exosome	1.4 (0.75–1.9)	ND	NA	0.62 (0.47–1.7)	0.28	1.2 (0.90–1.5)	0.77	NA	0.47
	**p-value**	0.53	NA		0.90		NA			
**Chemokines**										
RANTES	Plasma	7052 (2852–15233)	13365 (8947–15416)	0.37	5239 (2256–10194)	0.69	19997 (1152–46594)	0.63	0.99	0.94
	Exosome	530.8 (320.2–1744)	232 (149–871)	0.37	197.5 (121.7–1059)	0.16	1875 (1026–2781)	0.14	0.06	0.04
	**p-value**	0.001	0.007		0.0016		0.68			
MCP-1	Plasma	31 (19–87)	13. (5.01–23)	0.02	20 (18–38)	0.34	62.8 (36.3–126)	0.37	0.01	0.02
	Exosome	3. 8 (1.5–4.7)	0.74^a^	NA	4.0 (1.6–6.4)	0.72	3.56 (2.1–19)	0.99	NA	0.79
	**p-value**	0.0001	NA		0.0008		0.02			

Values are expressed as median (25^th^-75^th^ inter-quartile range)

^a^-detectable only in one sample

^b^-Comparison between HIV- positive non-tobacco smokers and HIV-positive tobacco smokers

^c^-Comparison between HIV-negative tobacco smokers and HIV-positive tobacco smokers, ND-not detectable; NA-not applicable

#### Anti-inflammatory cytokines

**Fig 5:**
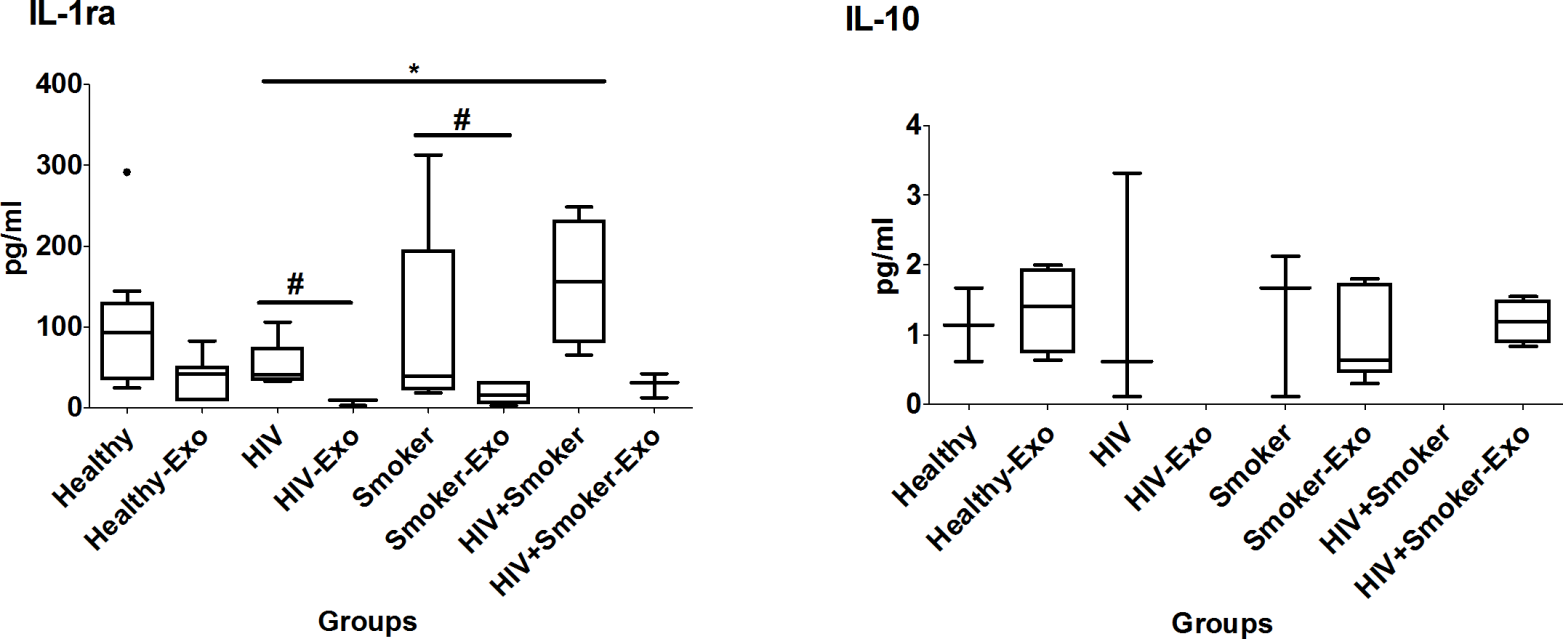
Box and whisker plots of anti-inflammatory cytokines between HIV-negative non-drug abusers (healthy), HIV-positive non-drug abusers (HIV), HIV-negative smokers (smokers) and HIV-positive smoker (HIV+smoker). For comparison with other study groups, the bar graphs for healthy and HIV subjects were replicated from Fig 2. The box represents the 25^th^-75^th^ quartile, the whiskers represent the range of values, the median is presented as a line inside the box, and the out-of-range values are presented as circles. The Mann–Whitney U test was used to compare between plasma and exosomes of different study groups as well as and significant p-values are represented as # (p≤0.05) and ## (p≤0.01). Comparison of cytokine levels among plasma as well as exosomes of different groups were done using the Mann–Whitney U test and significant p-values are given as * (p≤0.05), ** (p≤0.01) and $ (p≤0.05), $ $ (p≤0.01), respectively.

Similar to pro-inflammatory cytokines (Fig 2), the level of IL-1ra in exosomes was relatively lower than the respective plasma samples of smokers and HIV-positive smokers (Fig 5). However, IL-10 exhibited different characteristics than the other cytokines in terms of its relative level in exosomes compared to plasma. The median IL-1ra level in plasma was relatively higher in HIV-positive smokers than in healthy and smoker subjects, and significantly higher than HIV subjects. Interestingly, the level of IL-10 in exosomes was similar to that of plasma in healthy and smoker subjects, and it was completely packaged in exosomes of HIV-positive smokers (Fig 5, Table 2)

#### Chemokines

**Fig 6:**
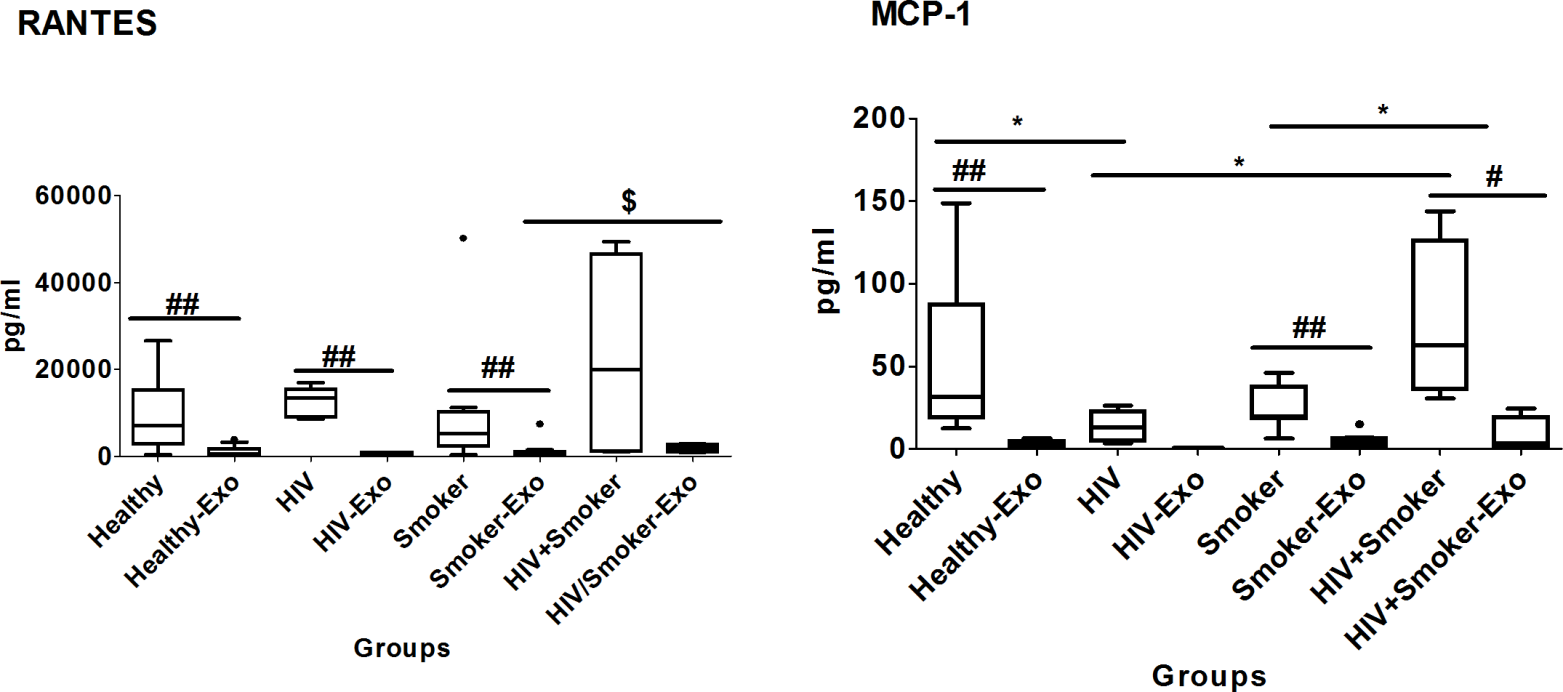
Box and whisker plots of chemokines between HIV-negative non-drug abusers (healthy), HIV-positive non-drug abusers (HIV), HIV-negative smokers (smokers) and HIV-positive smokers (HIV+smoker). For comparison with other study groups, the bar graphs for healthy and HIV subjects were replicated from Fig 3.The box represents the 25^th^-75^th^ quartile, the whiskers represent the range of values, the median is presented as a line inside the box, and the out-of-range values are presented as circles. The Mann–Whitney U test was used to compare between plasma and exosomes of different study groups as well as and significant p-values are represented as # (p≤0.05) and ## (p≤0.01Comparison of cytokine levels among plasma as well as exosomes of different groups were done using Mann–Whitney U test and significant p-values are given as * (p≤0.05), ** (p≤0.01) and $ (p≤0.05), $ $ (p≤0.01), respectively.

Similarly to the results presented in Fig 3, the chemokines RANTES and MCP-1 showed significantly higher levels in plasma than exosomes obtained from smokers and HIV-positive smokers. Importantly, the MCP-1 level in plasma of HIV-positive smokers was significantly higher than the HIV and smoker groups (Fig 6, Table 2). Eexosomes of HIV- positive smokers had significantly higher levels of RANTES than those of smoker or healthy subjects. Exosomal MCP-1 also showed a relatively increased level in HIV-positive smokers compared to both HIV and smoker subjects.

## Discussion

We conducted a cross-sectional study to determine the expression of cytokines and chemokines in the exosomes of HIV-infected individuals and/or drug abusers. We also studied the relative packaging of cytokines and chemokines into exosomes of HIV-positive and/or drug-abusing subjects. As the HIV-induced immunomodulation might be altered by alcohol and tobacco abuse, there is a need to identify a physiological marker to indicate the immune status of HIV patients with drug abuse disorders. The present study could potentially provide an important biomarker for HIV patients with altered immune function due to drug abuse. In the this study, we observed that some cytokines can be selectively packaged in exosomes, and their levels vary depending on the conditions of their exposure. This is the first study on the analysis of the relative levels of plasma exosomal cytokines and chemokines in HIV-positive drug abusers.

HIV infection is associated with increased expression of pro-inflammatory cytokines and chemokines [36,37]. In the present study, the cytokine/chemokine levels were relatively low in plasma samples from the HIV group relative to the healthy group, except for IL-6 and RANTES. Pro-inflammatory cytokines have been reported to be high in HIV-positive African women without an AIDS diagnosis, compared to those with AIDS [38], suggesting that pro-inflammatory cytokines could be involved only in the early phase of infection. Thus, the unaltered or decreased pro-inflammatory cytokines in our study population may indicate the stage of HIV progression to AIDS. Further, our samples were recruited from a region of Africa where access to ART is limited, [39] which could have led to the enrollment of subjects with an advanced stage of HIV infection. Our findings are concordant with Mack M et al study which reported high levels of IL-6 in HIV-infected patients [40].

Among the studied cytokines and chemokines, TNF-α (40%), MCP-1(5%), IL-1ra (14%), and RANTES (3%) were packaged into exosomes, in HIV-infected non-drug abusers. Literature reports suggest that exosomes facilitate HIV replication by mechanisms such as transfer of viral entry receptors (CCR5 and CXCR4) [41,42], miRNA [43,44], and viral proteins (Nef) [45,46]. However, the study of the role of exosomal cytokines in HIV replication is in its infancy. Exosomes can transport cytokine-inducing molecules such as HIV TAR RNA[47] and Nef protein [48] from HIV-infected cells, thereby increasing cytokine expression in recipient cells. Apart from cytokine-inducing molecules, exosomes are also reported to carry cytokines themselves [30,49], which may exacerbate HIV replication. Our findings are consistent with studies by Konadu et al. and Johnson et al., who showed that plasma exosomes of HIV-infected individuals carry cytokines [30,49]. Konadu et al. also reported that exosomes derived from HIV-seropositive patients had significantly higher levels of cytokines than the exosomes of HIV-seronegative patients [30]. However, in our study, there was no difference in exosomal cytokine levels between healthy and HIV groups, except IL6 and perhaps RANTES, which could be due in part to small sample size. Furthermore, the array of cytokines, that we have tested for is different from that considered by other studies.

Alcohol exposure regulates the production and function of signaling molecules that coordinate the immune response in alcoholic liver disease and influence the cross-talk between liver parenchymal and immune cells[50]. Interactions between immune cells can occur by direct cell-cell contact and/or by cytokine production. These cytokines can be transferred to distant cells via secretion into the plasma, or by exosomal transport. In the present study, IL-10 was not detectable in plasma samples but was found in the plasma exosomes of alcohol users. The percentages of the cytokines TNF-α, MCP-1, IL-8, IL-1ra, and RANTES packaged in exosomes, compared to plasma, were 28%, 18%, 75%, 14%, and 25%, respectively, indicating a potentially significant role of exosomes in mediating the effects of these cytokines on recipient cells. We have previously demonstrated that plasma derived exosomes provoke an inflammatory response in recipient mice *in vivo* [51]. Elevated levels of the pro-inflammatory cytokines TNF-α, IL-1β, and IL-8 have been observed in alcoholic liver disease patients[52]. Further, ethanol induces extracellular vesicle production and incubation of these vesicles with naïve monocytes results in the production of cytokines by the recipient cells [53]. IL-10 plays a role in reducing alcoholic liver injury and inflammation [54,55]. In the present study, complete packaging of IL-10 in exosomes derived from alcohol-drinking subjects may indicate the role of exosomes in mediating the protective effects of IL-10 against alcohol-induced liver injury. Our results support a finding where EVs derived from alcohol-exposed monocytes have been reported to stimulate IL-10 production in naïve monocytes [53]. Therefore, investigating the protective role of exosomal IL-10 in alcohol-exposed cells may provide insights in using exosomes as a therapeutic agents to reduce alcohol-induced liver injury. Further, exosomes can serve as biomarker of alcohol-induced liver injury as the exosome numbers were strongly correlated with a marker of liver injury, AST levels (r = 0.80), in the presence of alcohol [56]. Apart from carrying cytokines, hepatocyte-derived exosomes also carry cytokine-inducing agents to macrophages in the event of alcohol exposure, indicating the cross talk between hepatic exosomes and immune cells [57]. Therefore, exosomes may play an important role in alcohol-induced tissue damage by altering cytokine production.

Alcohol consumption increases susceptibility to HIV infection by affecting the nutrition and immunological functions of a person[58]. Further, acute or chronic alcohol consumption has been reported to increase HIV replication [59,60] and dysregulate cytokine production [61,62], it is also associated with poor adherence to ART therapy [63,64]. Alcohol abuse also causes neuro-inflammation [65,66] and increases the risk of secondary infections [67], thereby decreasing survival rates in HIV patients [68]. In our study, we investigated cytokine levels in exosomes of HIV-positive alcohol drinkers to find a potential marker to diagnose alcohol-induced toxicity in HIV patients. HIV-positive alcohol drinkers had significantly lower levels of exosomal IL-1ra compared to HIV-negative alcohol drinkers (p = 0.02; Table 1), and there was no change in levels when compared with the HIV-positive non-drinkers (Table 1). Similar to the alcohol group, IL-10 was completely packaged in exosomes of HIV-positive drinkers. Moreover, compared to plasma, the percentage of the cytokines TNF-α, IL-8, MCP-1, RANTES and IL-1ra, packaged in exosomes of HIV positive alcohol users are 15%, 10%, 14%, 14%, and 10% respectively. The cytokines packaged in exosomes are more stable than the circulating biological molecules[34] and can cross the blood-brain barrier [69,70]. Studying exosomal cytokines may reveal optimistic views towards diagnosis and therapeutic treatments for alcohol-induced toxicity in HIV patients.

All the studied cytokines were present in the plasma as well as the exosomes of smokers, except for IL-6, which was completely packaged in exosomes. Though not statistically significant, both plasma and exosomes from smokers had higher levels of IL-1β compared to those of healthy subjects. Cigarette smoking is associated with variations in production of inflammatory markers [71]. For example, increased levels of IL-1β and IL-1ra have been observed in bronchoalveolar lavage of smokers, whereas their levels were decreased in cancer patients who smoke [71]. Tobacco smoking status has been shown to affect plasma cytokine levels in different phenotypes of chronic obstructive pulmonary disease[72]. Therefore, cytokine expression varies based on the smoking status of an individual, which perhaps could have been contributed to differences in the outcome of studies. We have also studied the cytokine levels in the exosomes of smokers and found that IL-6 is completely packaged in exosomes. Further, relative to plasma, 11% of TNF-α, 42% of IL-1β, 18% of IL-8, 19% of MCP-1, 19% of IL-1ra, and 11% of RANTES were packaged in exosomes. Very few studies have been conducted on the role of exosomal cytokine levels in smoking-induced inflammation. Alveolar macrophages have been reported to release exosomes upon stimulation and these exosomes were shown to carry the protein suppressor of cytokine signaling 1 (SOCS1). Incubation of alveolar epithelial cells with exosomes derived from alveolar macrophages showed the uptake of exosomes containing SOCS1 protein and inhibit cytokine-induced signaling in these cells [73]. Further, the release of SOCS1-containing vesicles was significantly reduced in smokers compared with non-smokers [73], which suggests that impaired activity of SOCS1 may contribute to the inflammatory responses that are associated with tobacco smoke exposure. Moreover, the expression of IL-6 and TNF-α were negatively correlated with the expression of SOCS1 in macrophages upon cigarette smoke exposure [74].These findings suggests that exosomes can be used as a mediators to target cytokine signaling pathways in case of smoking-induced inflammation.

Smoking is also known to exacerbate HIV replication by various mechanisms. Cytokine levels were found to be altered in HIV-positive smokers compared to HIV negative smokers[14]. Smoking induces cytokine production, which is involved in HIV infection[75,76]. Interestingly, in our study, HIV-positive smokers had significantly higher levels of plasma IL-8, MCP-1, and IL-1ra compared to HIV-positive non-smokers. In addition, HIV-positive smokers had significantly higher levels of plasma MCP-1 compared to those of HIV-negative smokers. Exosomes of HIV-positive smokers contained IL-1β (3%), IL-8 (24%), MCP-1 (11%), IL-1ra (18%), and RANTES (8%). Interestingly, IL-6 and IL-10 were not detectable in exosomes of HIV patients but were present in exosomes of both HIV-positive and uninfected smokers. HIV-positive smokers had higher exosomal IL-6 than healthy subjects, and higher exosomal RANTES than HIV-negative smokers. RANTES causes resistance to HIV infection by downregulating the CCR5 receptor, a chemokine receptor and co-receptor for viral entry, there by blocking HIV [77–79]. Complete packaging of IL-10, an anti-inflammatory cytokine, and increased RANTES levels in HIV- positive smokers may indicate that smoking induces cytokines in exosomes, which reduce HIV replication. Recently, a study from our group has shown that exosomes derived from macrophages exposed to tobacco constituents protect against cytotoxicity and reduce HIV replication in macrophages [35]. Further investigation of the roles of exosomal contents derived from murine models of HIV and drug abuse may provide potential data on the *in vivo* role of these exosomal cytokines.

## Conclusion and future directions

The present findings suggest that plasma cytokines are packaged in exosomes. Increased cytokine levels in plasma as well as exosomes of HIV-positive drug abusers, when compared with HIV and drug abuse groups alone, suggest a novel mechanism of pathogenesis in cases of drug abuse and HIV comorbidity. Despite a relatively low number of subjects, the current study provides an evidence of the role of specific plasma exosomal cytokines and chemokines, which are specifically packaged in HIV-infected drug abusers compared with only HIV or drug abuse groups. This finding also provides important information on packaging of specific cytokines and chemokines under HIV and drugs of abuse conditions, which is an important step towards finding potential biomarkers for the interaction between HIV and drugs of abuse. We are in the process of recruiting large cohorts from these groups in the USA, which would provide further information about the role of plasma exosomal cytokines and chemokines in the interaction between HIV and drugs of abuse. We do expect that plasma exosomal cytokine levels would vary between mild-to-moderate and heavy drug abusers, and studying the relative exosomal cytokine levels in subjects with different degrees of drug abuse would provide valuable information about how it can affect the systemic immune response, and thus could be a promising avenue for future investigation.

## Supporting information

S1 Dataset Cytokine and chemokine levels in plasma and exosomes of HIV-neagtive non-drug abusers (Healthy), HIV-positive non-drug abusers (HIV), HIV-negative alcohol drinkers (alcohol), HIV-positive alcohol drinkers (HIV+alcohol), HIV-negative smokers (smoker), and HIV-positive smokers (HIV+smokers). (XLSX)
